# Can Non-lytic CD8+ T Cells Drive HIV-1 Escape?

**DOI:** 10.1371/journal.ppat.1003656

**Published:** 2013-11-14

**Authors:** Nafisa-Katrin Seich al Basatena, Konstantinos Chatzimichalis, Frederik Graw, Simon D. W. Frost, Roland R. Regoes, Becca Asquith

**Affiliations:** 1 Imperial College, London, London, United Kingdom; 2 Birkbeck University, London, United Kingdom; 3 Los Alamos National Laboratory, Los Alamos, New Mexico, United States of America; 4 University of Heidelberg, Heidelberg, Germany; 5 University of Cambridge, Cambridge, United Kingdom; 6 ETH, Zurich, Switzerland; Emory University, United States of America

## Abstract

The CD8+ T cell effector mechanisms that mediate control of HIV-1 and SIV infections remain poorly understood. Recent work suggests that the mechanism may be primarily non-lytic. This is in apparent conflict with the observation that SIV and HIV-1 variants that escape CD8+ T cell surveillance are frequently selected. Whilst it is clear that a variant that has escaped a lytic response can have a fitness advantage compared to the wild-type, it is less obvious that this holds in the face of non-lytic control where both wild-type and variant infected cells would be affected by soluble factors. In particular, the high motility of T cells in lymphoid tissue would be expected to rapidly destroy local effects making selection of escape variants by non-lytic responses unlikely. The observation of frequent HIV-1 and SIV escape poses a number of questions. Most importantly, is the consistent observation of viral escape proof that HIV-1- and SIV-specific CD8+ T cells lyse infected cells or can this also be the result of non-lytic control? Additionally, the rate at which a variant strain escapes a lytic CD8+ T cell response is related to the strength of the response. Is the same relationship true for a non-lytic response? Finally, the potential anti-viral control mediated by non-lytic mechanisms compared to lytic mechanisms is unknown. These questions cannot be addressed with current experimental techniques nor with the standard mathematical models. Instead we have developed a 3D cellular automaton model of HIV-1 which captures spatial and temporal dynamics. The model reproduces *in vivo* HIV-1 dynamics at the cellular and population level. Using this model we demonstrate that non-lytic effector mechanisms can select for escape variants but that outgrowth of the variant is slower and less frequent than from a lytic response so that non-lytic responses can potentially offer more durable control.

## Author Summary

The interplay between viruses and the immune system cannot always be studied with current experimental techniques or commonly used mathematical models. Consequently, many important questions remain unanswered. The questions we wished to address fall into this category. Recent evidence strongly suggests that CD8+ T cells control SIV, and potentially HIV-1, primarily by secreting anti-viral factors rather than by killing infected cells. However, this does not seem compatible with the common observation that HIV and SIV evolve to escape the immune response. Soluble anti-viral factors, like RANTES which protects uninfected cells from infection, would be expected to inhibit both wild-type and variant virus. Furthermore, the high speed and motility of T cells in lymphoid tissue will increase homogeneity and again decrease the likelihood that an escape variant can have a selective advantage in the face of non-lytic control. We wanted to understand whether viral escape is proof that HIV-1- and SIV-specific CD8+ T cells kill infected cells, determine the factors that facilitate viral escape, and investigate the comparative efficiency of lytic and non-lytic responses in controlling viral infections. Here we develop an elaborate but robust computational framework that captures T cell kinetics and spatial interactions in lymphoid tissue to addresses these important questions.

## Introduction

There is good evidence that CD8+ T cells control replication of human (HIV-1) and simian (SIV) immunodeficiency virus [1]. CD8+ T cells can control viral replication via lytic and non-lytic effector mechanisms. Lytic mechanisms are mediated by secretion of perforin and granzymes or stimulation of the Fas/FasL pathway and result in direct killing of the productively-infected cell. Non-lytic CD8+ T cell effector mechanisms are mediated by multiple soluble factors that can suppress viral production by infected cells or reduce the susceptibility of uninfected cells to infection [2]–[9]. The identity of these non-lytic factors remains controversial. Some studies [10]–[15], but not all [16], [17], have reported that the CD8+ T cell-secreted cytokine IFN-γ has a suppressive effect on HIV-1 (by upregulating MHC class I expression and inducing the expression of intrinsic defence factors including TRIM1α, APOBEC and tetherin). Similarly, chemokines such as RANTES, MIP-1α and MIP-1β which bind CCR5 and act as competitive inhibitors of CCR5-mediated HIV/SIV entry [18] are also thought to play a role, indeed polymorphisms in the RANTES promoter which increase mRNA transcription are associated with slower disease progression [19], [20]. However, whether CD8+ T cells secrete these chemokines in sufficient quantities has been disputed [21], [22]. Finally, CD8+ cell antiviral factor (CAF) is reported to inhibit HIV-1 replication by blocking transcription [23]–[25].

Recently, it was reported that, following CD8+ T cell depletion in SIV-infected macaques, viral load increased significantly, however the lifespan of SIV-infected cells was unaltered [26], [27]. These results led to the suggestion that SIV is controlled primarily via non-lytic mechanisms; a suggestion which was further studied and corroborated in [28]. However, the finding remains controversial [29]. Interestingly, a similar absence of correlation between the lifespan of productively infected cells and the strength of the immune response has also been reported in HIV-1 infection [30].

HIV-1 and SIV are characterised by the selection of viral mutants that can escape CD8+ T cell responses. The error-prone virus replication process results in frequent base substitutions (10^−4^–10^−3^ per bp per round of replication [31], [32]). When these mutations lie in or near CD8+ T cell epitopes they can impair peptide processing, presentation or T cell receptor binding resulting in reduced T cell recognition of the variant-infected cells. If the benefit to the virus of evading the CD8+ T cell response is not outweighed by impaired replication conferred by the mutation(s) then the variant strain will have a net growth advantage compared to the wild type strain and will typically outgrow the wild type. There are numerous studies reporting the outgrowth of HIV-1 and SIV variants that can escape CD8+ T cell control during primary [33]–[36] and chronic infection [37]–[40].

The observation of frequent HIV-1 and SIV escape poses a number of questions. Most importantly, if CD8+ T cells control SIV, and potentially HIV-1, primarily by non-lytic mechanisms then why is viral escape observed? Whilst it is clear that under a lytic mechanism an escape variant can have a fitness advantage compared to the wild-type strain, it is less obvious that this holds in the face of non-lytic control. Although non-lytic factors will be elicited specifically by wild type-infected cells, these factors will act non-specifically on both wild type and variant-infected cells. Consequently, escape variants will not necessarily have a selective advantage. Indeed, in a well-mixed population, non-lytic CD8+ T cells cannot select for escape variants. The high motility of T cells in the lymph node and spleen, as measured by intravital two photon microscopy [41]–[43], as well as the ability of soluble factors to diffuse over large distances [44], will tend to destroy local effects, increasing homogeneity and decreasing the probability that soluble factors can drive HIV-1 and SIV escape. Is the consistent observation of viral escape (i.e. selection of escape variants) proof that SIV and HIV-1-specific CD8+ T cells lyse infected cells? Additionally, the rate at which the variant strain replaces the wild type strain is used as a surrogate for the strength of the CD8+ T cell response assuming that this response is lytic [45]–[50]. The relationship between the rate of escape and the strength of the CD8+ T cell response is unclear when the CD8+ T cell response is primarily non-lytic and may fundamentally change experiment interpretation. Finally, the potential anti-viral efficiency of non-lytic mechanisms compared to lytic mechanisms is unknown.

To investigate the dynamics of non-lytic HIV-1-specific CD8+ T cell responses it is essential to consider *in vivo* spatial as well as temporal kinetics. This is not possible with current experimental techniques nor with the standard ordinary differential equation-based viral dynamics framework which assumes spatial homogeneity. We therefore developed an agent-based *in silico* approach. A similar approach has been successfully used to investigate T and B cell dynamics [51]–[54]. Using this approach we investigate whether non-lytic CD8+ T cell responses can select for HIV-1 escape variants; additionally, we identify factors involved in variant outgrowth and quantify the relationship between viral control and the outgrowth rate.

## Results

### A spatial model of HIV-1 dynamics

**Figure 1:**
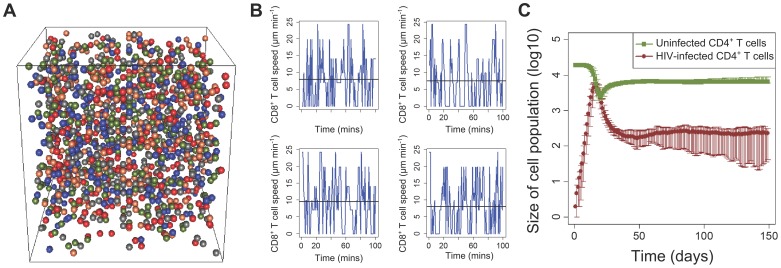
The cellular automaton model accurately reproduces T cell and viral dynamics at the cellular and population level. (a) A snapshot of the 3D cellular automaton model. The different coloured nodes represent the different cell populations. (b) The speed of four individual simulated CD8+ T cells in the CA model as a function of time. The mean speed is 9 µm/min and the mean motility coefficient is 75 µm^2^/min. (c) Dynamics of uninfected and infected CD4+ T cells over a course of 150 days. The bars represent the 95% central range values.

We developed a Cellular Automaton (CA; Figure 1a) to simulate HIV-1 infection in a small volume of the spleen. A CA is a computer simulation of a system in which cells move in space and time on a three-dimensional lattice.

We model 6 cell populations: uninfected CD4+ T cells, CD4+ T cells infected with the wild type strain of HIV-1, CD4+ T cells infected with an escape variant strain of the virus, CD8+ T cells which are specific for a single HIV-1 epitope, macrophages, and a generic splenocyte population. The reticular network of the spleen is also modelled (see Methods for further details). The model was parameterised by experimental observations. When parameter estimates varied in the literature we ran the simulations with a range of parameter values to check the robustness of our conclusions.

We explicitly model a single virus-specific CD8+ T cell response (i.e. clone or clones recognising a single epitope). Initially, we assume that this CD8+ T cell response controls virus infection by lysing infected cells (“lytic response”). This is later generalised to consider non-lytic effector mechanisms (“non-lytic response”). Infected cell death attributable to all other mechanisms (including other virus-specific CD8+ T cells, activation induced cell death and virus-induced cytopathicity) is treated as a constant. In chronic HIV-1 and SIV infection, under the assumption that CD8+ T cells control virus infection via a lytic mechanism, a single CD8+ T cell response has been estimated to kill productively infected cells at a rate of 0.01–0.05 per day [47]–[49]. We tune the model to give CD8+ T cell killing rates in this range. If a virus-specific CD8+ T cell neighbours an infected CD4+ T cell then three model parameters influence the infected cell death rate: 1) the probability of recognition (i.e. the probability that the CD8+ T cell successfully identifies that its neighbour is infected), 2) the scanning time (i.e. the time to make this decision) and 3) the duration of the killing process. We found that the CD8+ T cell killing rate is only weakly dependent on the scanning time and the duration of killing (Figure S1 in Text S1). We therefore control the killing rate by adjusting the probability of recognition. Because of the high motility of T cells the frequency with which CD8+ T cells neighbour infected cells is high and so only a low probability of recognition is required to yield a plausible population killing rate (a probability of recognition of 0.001 to 0.003 gives a mean population killing rate of 0.01 per day to 0.05 per day). This probability of recognition seems unexpectedly low. It may be that the probability of recognition of naturally infected cells, which has not been estimated experimentally, is genuinely low (perhaps because of down-regulation of class I molecules from the infected cell surface or other immune escape mechanism). Indeed, even for peptide-pulsed splenocytes which would be expected to be easily recognised, a large proportion of encounters between effectors and targets do not result in lysis [55]. The only way in which the probability of recognition of naturally infected cells could be increased and still give a plausible rate of CD8+ T cell killing, is if the motility or frequency of specific CD8+ T cells is much lower than observed. Decreasing the motility or frequency of specific CD8+ T cells (with a corresponding increase in the probability of recognition) will not change the frequency of successful contacts between effectors and targets and so it would not alter the model conclusions. Conclusions were also robust to an increase in the killing rate via an increase in the probability of recognition. Finally, the killing rate did not change significantly if the grid size was increased (data not shown).

### The CA model reproduces *in vivo* dynamics at the cellular and population level

The speed, motility coefficient and displacement of T cells has been measured experimentally using two-photon microscopy under different experimental settings in multiple studies [43], [56]–[60]. The average speed of non-activated T cells is reported to be 10–15 µm/min with a maximum of 25 µm/min [43], [58], the motility coefficient is 50–100 µm^2^/min and the displacement is observed to be proportional to the square root of time (i.e. consistent with a random walk). Most of these measurements have been made in the lymph nodes; for the spleen, they are suggested to be comparable but lower [61], [62]. In our model simulations, prior to infection, the mean CD4+ and CD8+ T cell speed is approximately 9 µm/min with a maximum of 25 µm/min (Figure 1b) and the average T cell motility coefficient is 75 µm^2^/min. Additionally, the displacement is proportional to the square root of time (Figure S2 in Text S1). As expected, the mean CD8+ T cell speed is reduced when infected cells are introduced in the model because of conjugate formation (Figure S3 in Text S1). The model thus reproduces the motility of T cells at the cellular level [41]–[43], [56], [61].

The CA model also reproduced cell and virus dynamics at the population level. Simulated infections displayed a typical viral expansion phase, with a viraemic peak reached after two to three weeks (Figure 1c). The infected CD4+ T cells in the first days post infection (dpi) grow at a rate of 1.5 d^−1^ which is within the 1–2 d^−1^ range that has been reported [63]–[65]. Close to the peak there is a large depletion of CD4+ T cells and the availability of uninfected target cells becomes limiting. The CD8+ T cell response which was introduced at day 10 post infection together with the target cell limitation result in the slowing down of viral growth and the subsequent decline to a viral set-point within a few weeks. The proportion of infected CD4+ T cells at the steady-state (>30 dpi) is of the order of 10^−3^–10^−2^, consistent with experimentally-observed values [66] .

### Non-lytic responses can drive viral escape

We used the CA to simulate lytic and non-lytic CD8+ T cell effector mechanisms. We divide non-lytic effector mechanisms (mediated by soluble factors secreted by CD8+ T cells) into those that protect uninfected cells from becoming infected and those that reduce viral production by infected cells. CD8+ T cells mediate non-lytic control via a number of soluble factors. As a case study, we model RANTES [67]. We assume that RANTES binds to all uninfected CD4+ T cells that travel through the area of secretion around the activated HIV-1-specific CD8+ T cells. This effect lasts for 10 hrs (as measured in [67]) and protects these uninfected targets from infection. Once this period expires the formerly protected cells become susceptible to infection. The *in vivo* area of diffusivity of RANTES is poorly quantified, we therefore consider a range of possible values: polarised secretion, and diffusive secretion with different radii (see Methods). In a similar way, a non-lytic effect that reduces virion production can be modelled by abrogating, for a given time, the infectivity of infected cells that cross the area of secretion. All other parameter values are identical between all the models; this allows us to study the different control mechanisms under the same conditions. We introduce an escape variant-infected population at 50 dpi.

As expected, lytic CD8+ T cells reproducibly drove viral escape (i.e. selected for the escape variant). The probability of fixation of the variant strain ranged from 67%–100% as the rate of killing varied from 0.01 d^−1^ to 0.05 d^−1^. The mean rate of viral escape (from introduction of the variant) varied from 0.03 d^−1^ to 0.07 d^−1^ over the same range. This agrees well with the experimentally observed rates in chronic infection which vary between 0.01 d^−1^ and 0.1 d^−1^ [35], [39], [49], [68].

**Figure 2:**
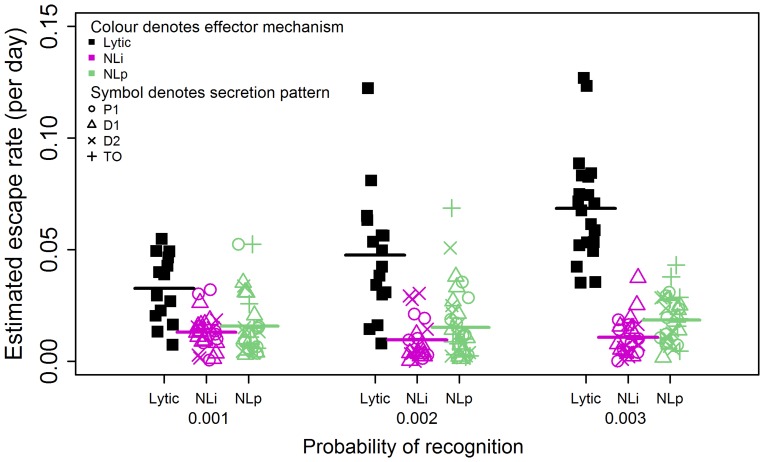
Non-lytic CD8+ T cells reproducibly drive viral escape but escape is slower than that driven by lytic CD8+ T cells. This was observed both for non-lytic factors that reduce infection and non-lytic factors that reduce production; for the three different probabilities of target cell recognition considered and for all secretion patterns considered. Abbreviations: NLi: Non-lytic model - reducing infection of uninfected CD4+ T cells, NLp: Non-lytic model - reducing virion production, TO: target cell only affected, P1: Polarised secretion (r = 1), D1: Diffusive secretion (r = 1) and D2: Diffusive secretion (r = 2). Different colours represent different effector mechanisms (black: lytic, pink: NLi, green: NLp) and different symbols represent different patterns of secretion (open circle: P1, open triangle: D1, x: D2, cross:TO). The horizontal bars represent the mean. Lytic CD8+ T cells drive escape significantly faster and more frequently than CD8+ T cells operating via a non-lytic mechanism which impairs viral infection or a non-lytic mechanism that impairs virion production. Mean rates of escape from lytic CD8+ T cells: 0.033 d^−1^, 0.048 d^−1^, 0.069 d^−1^ for Pr = 0.001, Pr = 0.002, Pr = 0.003 respectively. Mean rates of escape from non-lytic CD8+ T cells that impair infection: 0.013 d^−1^, 0.010 d^−1^, 0.011 d^−1^ for Pr = 0.001, Pr = 0.002, Pr = 0.003 respectively. Mean rates of escape from non-lytic CD8+ T cells that impair virion production: 0.016 d^−1^, 0.015 d^−1^, 0.018 d^−1^ for Pr = 0.001, Pr = 0.002, Pr = 0.003 respectively. In every case the difference between lytic and non-lytic escape rates is significant (P<0.001, Wilcoxon Mann Whitney test two-tailed). The frequency of fixation is given in Table 1.

We found that when CD8+ T cell controlled viral infection via a non-lytic mechanism, either by reducing infection or by reducing viral production, then the selection of escape variants was still repeatedly seen. However fixation was less frequent (Table 1) and the rate of escape was lower (Figure 2) than in the lytic case (see next section).

**Table 1:** The percentage of simulations that result in variant fixation.

Pr	Models							
	Lytic	Non lytic (impair infection)			Non lytic (impair production)			
	-	P1	D1	D2	TO	P1	D1	D2
0.001	67%	43%	67%	24%	50%	57%	65%	43%
0.002	76%	29%	43%	38%	64%	57%	79%	64%
0.003	100%	48%	48%	38%	64%	64%	42%	64%

Abbreviations: Pr = Probability of recognition by CD8+ T cells, TO = Target Only, P1 = Polarised secretion (r = 1), D1 = Diffusive secretion (r = 1) and D2 = Diffusive secretion (r = 2).

We varied the model parameters to ascertain how robust the observation of escape from non-lytic factors was. Firstly, we varied the pattern of soluble factor secretion from polarised (affecting the 9 cells on the nearest grid face) to diffusive with a radius of one, two, four or five cells (affecting the 26, 124, 728 or 1330 nearest neighbours respectively). It is unlikely that RANTES (and other soluble factors that bind to CD4+ T cells) would travel further than this without being bound given the high density of cells in the spleen [56], [69]. We also varied the probability of CD8+ T cell recognition, the frequency of specific CD8+ T cells, the half-life of the soluble factor, the volume of the spleen modelled, the distance that free virus travels and the initial frequency of escape variant-infected cells (data not shown). In every case escape from non-lytic CD8+ T cells remained a frequent occurrence. We therefore conclude that the observation of viral escape is not incompatible with a CD8+ T cell response that controls infection entirely via a non-lytic mechanism.

**Figure 3:**
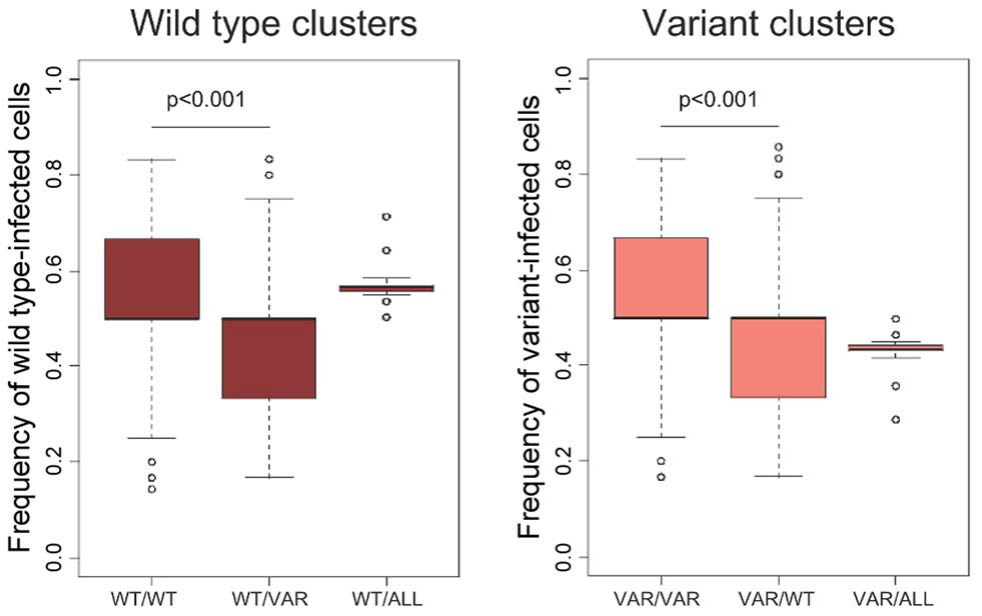
Existence of clusters of wild type and variant-infected cells. The percentage of wild type-infected cells neighbouring a wild type-infected cell is significantly higher than the percentage of variant-infected cells. The same is true for variant-infected cells. We track simulated wild type and variant-infected CD4+ T cells (≈500 cells) for multiple timepoints. Abbreviations: WT/WT = Wild type infected cells in the immediate area (r = 1, i.e. 26 nodes considered) surrounding a wild type-infected cell, WT/VAR = Wild type infected cells in the immediate area surrounding a variant-infected cell, WT/ALL = Wild type-infected cells on the whole grid. VAR/VAR, VAR/WT and VAR/ALL are defined similarly.

**Figure 4:**
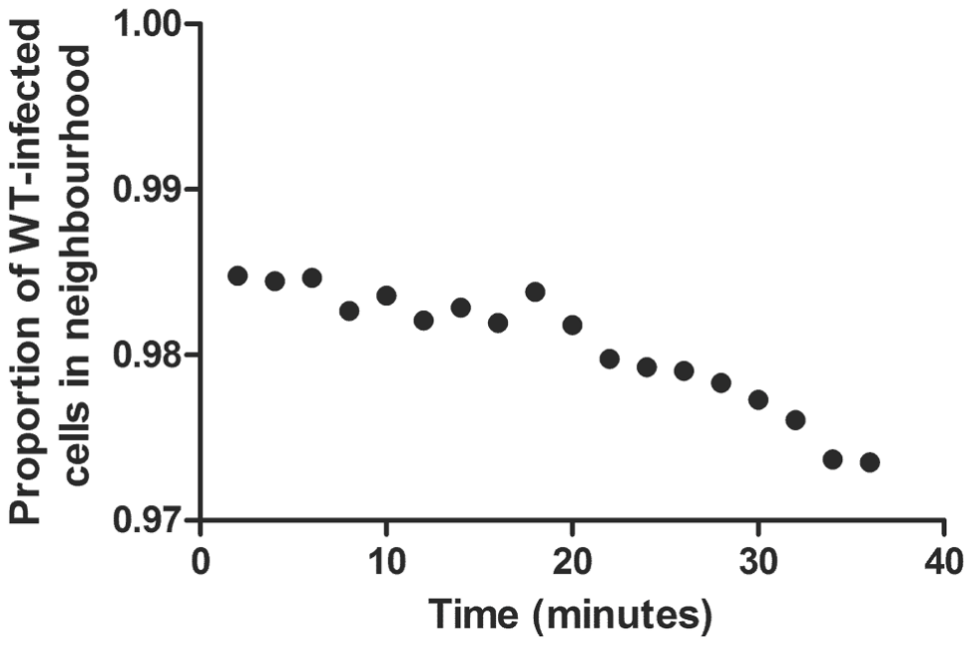
Infected cells in the neighbourhood of CD8+ T cells secreting non-lytic factors. The mean fraction of wild type-infected cells neighbouring secreting CD8+ T cells was evaluated over time. We tracked the proportion of wild type- and variant-infected cells in the immediate neighbourhood (radius = 1, i.e. 26 neighbours) of 5,425 secreting CD8+ T cells. In these runs, CD8+ T cells secreted non-lytic factors for 30 mins after triggering and were tracked for 34 minutes. The proportion of wild type-infected cells was high (i.e. the majority of affected cells were wild-type infected, conferring an advantage upon variant-infected cells), but not 100% (i.e. some variant-infected were also affected by the factor) and the proportion of wild type-infected cells decreased significantly over time (i.e. the spatial heterogeneity that conferred an advantage upon escape variants was short lived). The correlation between the proportion of wild type-infected cells and time during the 30 mins post CD8+ T cell-triggering was significant, Spearman correlation coefficient = −0.93, p<10^−6^.

The non-lytic CD8+ T cell effector mechanisms affect both wild type and escape variant populations; so under the assumption of spatially well-mixed cell populations, the escape variant would not be expected to have an advantage. We explored whether there are spatial patterns that can explain the observed advantage of the variant population under a non-lytic control mechanism. Although the median frequency of infected cells neighbouring a wild type- and variant-infected cell was similar, the overall distribution is highly skewed. A wild type-infected cell has a significantly higher frequency of wild type-infected than variant-infected cells in its immediate neighbourhood (p<0.001, two-tailed,Wilcoxon rank sum test) and likewise for a variant-infected cell (Figure 3). To investigate the evolution of this spatial heterogeneity over time we tracked the neighbourhoods of CD8+ T cells following triggering by wild type-infected cells. The proportion of infected cells in the neighbourhood that were infected with wild type and variant virus was recorded for the duration of secretion of the non-lytic factors. This analysis (Figure 4) showed that the majority of cells that would be affected by the secreted factors were indeed wild type infected, confirming our finding that the heterogeneity offers the variant a selective advantage. However, variant-infected cells were also present in the neighbourhood and their frequency increased significantly over time (p<10^−6^, two-tailed, Spearman correlation coefficient = 0.93).

It has been observed that the rate of escape is significantly more rapid in acute than in chronic infection [45], [47], [49]. During acute infection the rate of escape is up to 0.4 per day with a median of 0.25 per day [47]. We investigated whether, during the acute phase, lytic and non-lytic responses could drive escape at such rapid rates. We found that seeding the variant during the acute phase (15 dpi) doubled the rate of escape from lytic responses but had little impact on the rate of escape from non-lytic responses. Consequently, escape from lytic responses was in the observed ranges but escape from non-lytic responses was not. Even in the extreme case, chosen to favour rapid escape, of a high probability of recognition (Pr = 0.003) and a polarised secretion pattern the maximum rate of escape from a non-lytic response was only 0.05 per day. This indicates that, at least during acute infection, HIV-1-specific CD8+ T cells that operate via a lytic mechanism are likely to be present (it does not rule out the possibility that non-lytic CD8+ T cells are also present at this time).

### Viral variants that evade non-lytic CD8+ T cell responses have a slow rate of escape

**Figure 5:**
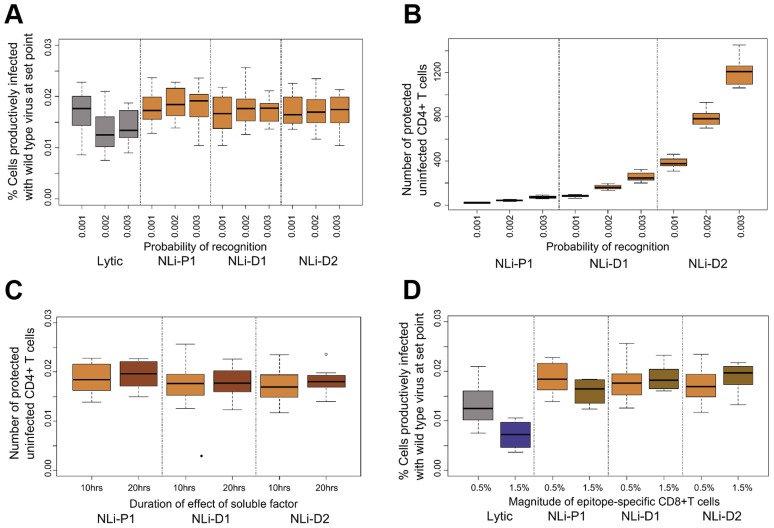
The immune control exerted by lytic and non-lytic CD8+ T cell responses. (**A**) The frequency of productively infected cells at setpoint for varying probability of CD8+ T cell recognition and for different secretion patterns. As the probability of recognition increases, the frequency of infected cells decreases for the lytic response but remains approximately constant for the non-lytic response for all secretion patterns. When Pr = 0.001 (first box) the frequency of infected cells is similar for the lytic and non-lytic responses (**B**) Number of uninfected CD4+ T cells protected from infection by a non-lytic CD8+ T cell response. The number of cells protected increases significantly as the probability of recognition and the area of diffusion increases (cumulative number by 50 dpi). (**C**) Set-point of productively infected cells when varying the duration of the protective effect of the soluble factor. Number of infected cells at setpoint does not decrease when the duration of effect is increased even though the number of cells protected robustly increases (**D**) Set-point of productively infected cells for different sizes of the epitope-specific CD8+ T cell clones. Here we show the results for the non-lytic control that blocks viral infection, similar results were found for non-lytic control that blocks viral production (Figures S6, S7 in Text S1 and data not shown). Results for more extensive parameter combinations are shown in supplementary information (Figures S10 and S11 in Text S1). The percentage of productively infected cells is calculated from 40–50 dpi. Abbreviations: NLi: Non-lytic model - blocking infection of uninfected CD4+ T cells, P1 = Polarised secretion (r = 1), D1 = Diffusive secretion (r = 1) and D2 = Diffusive secretion (r = 2).

We find that viral escape from a non-lytic response is slower than escape from a lytic response (Figure 2). We investigated whether this slower escape can be attributed to weaker anti-viral control by a non-lytic response. For the lytic response, as the probability of recognition was increased the anti-viral control exerted by lytic CD8+ T cells increased; i.e. the number of new infections prevented increased and the frequency of infected cells at set point decreased (Figure 5A, Figure S4 in Text S1). As expected both the rate of escape and the probability of variant fixation increased proportionally (Figure 2, Table 1 & data not shown). Under the same conditions, for the non-lytic response that decreased infectivity, we found that as the probability of recognition or area of secretion was increased there was a substantial increase in the number of uninfected cells that were protected (Figure 5B) but that, in marked contrast, the anti-viral control was unchanged; i.e both the number of new infections prevented (Figure S5 in Text S1) and the frequency of infected cells at setpoint remained approximately constant (Figure 5A). For the minimum probability of CD8+ T cell recognition considered (Pr = 0.001), anti-viral control (both the number of infections prevented and the frequency of infected cells at setpoint) was similar between the lytic and non-lytic model of the CD8+ T cell response (Figure 5A) and so at this point a fair comparison of the escape rates could be made. Despite the similar anti-viral control, lytic CD8+ T cells still drive escape significantly faster (approx. 0.033 per day vs 0.013 per day, p<0.001; Figure 2) and more frequently (67% v 43%, p<0.05; Table 1) than CD8+ T cells operating via a non-lytic mechanism which impairs viral infection. Repeating the simulations for a non-lytic mechanism that reduces virion production yielded similar results (Figures S6, S7, S8 in Text S1). The rate of viral escape, in the case where variant fitness costs are low, is used to compare the magnitude of anti-viral control [45]–[50]. Our results suggest that this approach is valid for comparisons between lytic responses but cannot be used to compare lytic with non-lytic responses as the same degree of anti-viral control results in significantly different escape rates.

### Antiviral control mediated by non-lytic effector mechanisms is difficult to enhance

HIV-1 escape from CD8+ T cells has been implicated in disease progression and in the failure of T cell-based vaccines in animal models [40], [70]–[72]. The observation that, despite comparable anti-viral control (as measured by the number of new infections prevented or by the frequency of infected cells at setpoint), non-lytic CD8+ T cell responses drive viral escape less frequently and less rapidly than lytic CD8+ T cell responses makes them an attractive target for boosting to enhance durable anti-viral control. We therefore examined what qualities of a non-lytic response were associated with anti-viral control.

There are four main quantities that could potentially strengthen the non-lytic response: 1) the radius of secretion, 2) the probability of recognition, 3) the duration of the effect of the soluble factor and 4) the size of the CD8+ T cell population. We already found that increasing the first two parameters did not substantially increase the anti-viral control (Figure 5B). Additionally, when we double the duration of the effect of the soluble factor from 10 to 20 hrs, we find no consistent substantial impact on anti-viral control either for CD8+ T cell secreted factors that block infection (Figure 5C) or block viral production (data not shown). Finally, we increase the size of the effector population (specific for a single epitope) from 0.5% to 1.5% of the splenocytes. Although, the number of cells that are protected by the soluble factors increases (Figure S9 in Text S1), again we find no significant increase in the anti-viral effect either in terms of new infections prevented (data not shown) or on the set-point of the infected cell population (Figure 5D). This result is in stark contrast to lytic control where the same increase in the probability of recognition or in the size of the effector population results in substantially better anti-viral control (Figure 5D, Figure S4 in Text S1). To investigate whether the anti-viral control conferred by non-lytic CD8+ T cells is bounded (i.e. cannot be increased above the observed value) we increased the model parameters substantially (beyond the physiological range). Under these circumstances, in some cases, we did observe significant improvement in anti-viral control. It was apparent that non-lytic responses that reduce infectivity were particularly hard to boost (Figure S10 and S11 in Text S1). Our findings taken together suggest that, whilst not impossible, it is difficult to enhance the anti-viral control conferred by non-lytic CD8+ T cell responses.

## Discussion

CD8+ T cells can control HIV viral burden through multiple mechanisms [9], [18], [26], [27], [73] which can be broadly classified as lytic and non-lytic. Although the existence of non-lytic CD8+ T cell-secreted factors in HIV-1 infection is shown by multiple studies (reviewed in [5]), little is known about the dynamics or *in vivo* relevance of such a response. Quantifying the CD8+ T cell non-lytic immune control requires information about spatial and temporal aspects of the system that cannot be captured with current *in vivo* experimental techniques. Instead we address these questions *in silico* by constructing a 3D cellular automaton model of HIV-1 dynamics parameterised by experimental data. Our aim is two-fold: 1) to investigate whether non-lytic CD8+ T cells can drive viral escape and 2) to explore the properties of non-lytic control.

We found that, for a wide range of model assumptions, both a non-lytic control mechanism that blocks viral infection (e.g RANTES) or restricts viral production (e.g. IFN-γ) reproducibly drives viral escape (Figure 2). Given that soluble factors diffuse and T cell motility is rapid this result is highly non-intuitive. We found localised clusters of wild type and variant-infected cells (Figure 3) that could enable viral escape since anti-viral factors elicited by wild type-infected cells would be more likely to affect their neighbours (other wild type-infected cells) and thus the variant-infected cells would have an advantage even though they are equally susceptible to non-lytic factors. Tracking this process over time (Figure 4) confirmed that the majority of infected cells in the vicinity of a secreting CD8+ T cells are indeed infected with wild type virus but that this proportion decreases significantly over time. We conclude that the variant has an advantage but this advantage is limited because the fast timescale of T cell motility destroys spatial heterogeneity. However, this is a dynamic process and the repetitive formation of such heterogeneous clusters is likely to provide a net advantage to the variant-infected cell population that can support its outgrowth.

We find that, when the lytic and non-lytic immune pressures are comparable in terms of number of new infections prevented and set-point proviral load, non-lytic responses drive escape significantly more slowly and less frequently than lytic responses (Figure 2). Consequently, the rate of escape cannot be used as a surrogate for the degree of antiviral control when comparing lytic and non-lytic effector mechanisms as the same control mediated via different effector mechanisms results in different rates of escape. At first sight, the observation that non-lytic CD8+ T cells drive escape slowly is reminiscent of a finding in [47] based on a theoretical model that assumes spatial homogeneity and thus has to impose escape externally. However, the two results are distinct, in the latter case the decreased rate of escape is attributable to strong CD8+ T cell control relative to the rate of viral replication rather than spatial factors.

It has been observed that the rate of escape during chronic HIV-1 infection is significantly slower than during acute infection [45], [47], [49]. We found that, when modelling chronic infection, the predicted rate of escape of variants escaping both lytic and non-lytic responses was broadly comparable with that observed experimentally. However, when simulating viral escape dynamics during the acute phase we found that it was impossible for a non-lytic (but not a lytic) response to drive escape at a rate comparable with experimental observation. This indicates that at least during acute infection, HIV-1-specific CD8+ T cells may operate predominantly via a lytic mechanism. It would be interesting to test this hypothesis by repeating the CD8+ T cell depletion experiment [26], [27] during acute infection. The reason behind the slower rate of escape in chronic infection is unknown. One possible mechanism, highlighted by this study, is a shift from a response dominated by lytic CD8+ T cells to one dominated by non-lytic CD8+ T cells.

Our findings have implications for studies of HIV progression and vaccine development. The appearance of escape mutants has been associated with loss of viral control and disease progression [70]; though there are numerous cases where escape does not result in progression or where progression occurs in the absence of escape [74], [75]. Our results indicate that, even for matched levels of antiviral control, non-lytic responses drive escape more slowly and less frequently than lytic responses (Figure 2), offering the potential of durable anti-viral control with less risk of viral escape. Furthermore, a non cytolytic effector mechanism has other advantages: it can act on multiple targets simultaneously, it can suppress infection without eliminating potentially vital cell populations and it can exhibit a ‘bystander’ protective effect [5]. Therefore, it can be argued that the tipping of balance towards non-lytic responses can be a strong weapon in the fight of HIV infection.

However, we find that it is difficult to enhance the efficiency of a non-lytic response. An increase in the probability of target cell recognition, the frequency of CD8+ T cells, the duration of the effect of soluble factors or the area of diffusion (within the physiological range) all failed to lead to better anti-viral control (Figure 5). In contrast a lytic CD8+ T cell response was easily “boosted”. This implies that even if many uninfected cells are protected from infection or the infectivity of many infected cells is reduced, if the infected cells are not eliminated, there are still enough available targets to sustain the set-point viral burden. Importantly, the difficult of boosting non-lytic responses is a function of the ratio of infected to uninfected targets. During *in vitro* suppression assays, where the majority of potential targets are infected, it is therefore likely to be easier to enhance non-lytic responses. However *in vivo*, where infected cells are a small minority, potentially very high numbers of effector CD8+ T cells are needed for efficient non-lytic suppression since even in theory it proved difficult to enhance a non-lytic response under conditions that readily enhanced lytic control.

In summary, this model provides a new framework for investigating CD8+ T cell control in HIV-1 infection. We find that, for comparable levels of anti-viral control, non-lytic responses select for escape variants less frequently and more slowly than lytic responses. However, anti-viral control mediated by a non-lytic response was difficult to enhance. Most importantly we repeatedly and reproducibly found that over a wide range of parameters, non-lytic CD8+ T cells were able to select for escape variants despite rapid T cell motility and soluble factor diffusion. We conclude that the observation of viral escape does not constitute proof that CD8+ T cells kill virus-infected cells in SIV and HIV-1 infection.

## Methods

The CA lattice consists of nodes and edges and is updated at every timestep (30 sec). Each node of the lattice represents a cell or part of the cell and has 26 neighbours. The grid size is set to 125,000 cells in most simulations (roughly 0.005–0.5% of the splenic volume); however, we do vary the grid size in order to explore if it affects our conclusions. The CA lattice is governed by toroidal boundary conditions where a cell leaving one side of the lattice reappears on the opposite side.

### Cell sizes

Most cells are represented by one node on the lattice, the exception is macrophages which occupy four nodes; this is to reflect the difference between the diameter of T cell which is 7 µm and that of macrophages which is calculated to be 10–16 µm and has been implemented as such in [53]. An additional set of nodes represents the reticular network, a rigid cellular structure found in the spleen. These nodes are immobile and act as spatial obstacles to the overall movement of cells. Only a small percentage (approx. 1%) of nodes is considered to be unoccupied since the spleen is a dense organ. The nodes that are left after setting all frequencies of the specific cell populations within biological ranges (Supplementary Methods in Text S1) are considered to be unspecified splenocytes. The cellular automaton is implemented in C++.

### Model assumptions

Modelling the dynamics of HIV-1 infection demands the incorporation of many complex rules. However, focusing on the specific questions that we are aiming to address and in order to speed-up the simulations and ease the interpretation of our results, we allowed for multiple assumptions to be included in the model as long as these would not alter the outcome of the tested hypotheses. The following assumptions were made:

1. The same motility parameters apply for all cell types since we focus on T cells for which the parameters have been experimentally defined in multiple studies [43], [56]–[60] .
2. We keep the density of the reticular network constant since it has been shown that it does not substantially effect cell-cell interactions [76].
3. Free virus is not explicitly included in the model. Infection (via free virions or cell-cell transmission) is simulated via a probability of infection of uninfected cells neighbouring a productively infected cell. This probability is set based on estimates of the HIV viral reproduction rate [65].
4. We do not make any distinction between activated and non-activated CD4+ T cells.
5. We explicitly model a CD8+ T cell response specific for a single HIV-1 epitope. Because we focus on the dynamics of infection after set point viral load we do not consider proliferation or contraction of this CD8+ T population.
6. There is no fitness cost associated with the escape mutations. Fitness costs will act to slow the rate and frequency of escape. There is evidence suggesting that, in many cases, this fitness cost can be low [49], [77].
7. We do not consider superinfection of infected cells. Findings regarding superinfection remain controversial in the literature [78], [79].

### CD4+ T cell proliferation

We define the influx of CD4+ T cells using *in vivo* proliferation rate estimates obtained with deuterated glucose in 7 treatment-naive HIV+ subjects [80] . The authors estimate that the mean CD4+ proliferation rate in HIV+ subjects is 0.025 d^−1^ when their mean CD4+ T cell count is approximately 400 µl^−1^, i.e. about 33% of a normal count. As an estimate of the proliferation rate we use a Hill function where j is calculated such that the probability of entering the grid, p_influx_ when the CD4+ T cell count is decreased by 67% provides a CD4+ T cell proliferation rate of 0.025 d^−1^ and n is chosen to provide a plausible change of p_influx_ with respect to the percentage of CD4+ T cells, U, at any given timepoint. We note that 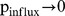 when CD4+ T cell count goes to normal, i.e. 100% of the initial population. This formulation simulates a homeostatic influx mechanism for the replenishment of CD4+ T cells which allows for a sustained viral infection in the CA model.
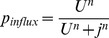



The probabilities which define whether a CD4+ T cell that entered the grid will be uninfected or infected (with the wild type or the variant strain) depend on the relative sizes and lifespans of these populations (see Supplementary Methods in Text S1). The new CD4+ T cells introduced in the grid appear at random free space cells as in [81].

### Virus reproduction and death

#### Reproductive rate

In the context of within host viral infection, the basic reproductive ratio, R_0_, is defined as the number of secondary infected cells produced by a primary infected cell during its lifetime assuming that uninfected target cells are not limiting. We consider *R*_0_ = 6 in accordance with estimates reported in the literature [65], [82]. At this viral reproduction rate, we record a peak in infected cell population 15–20 *days* post infection (dpi). HIV-1 infection of new cells is thought to occur over a relatively short radius [83]–[85]; this is probably attributable to a number of factors including high uninfected cell density in the lymphoid tissues (meaning viral particles will immediately attach to nearby cells), inherent instability of HIV-1 virions [86], [87] rapid clearance of virions *in vivo* [88], [89] as well as direct cell-to-cell transmission [90]–[94]. Accordingly, we model the spread of infection (mediated by free virions and cell-cell transmission) by allowing all cells neighbouring a productively infected cell (26 in total, r = 1) to have a probability of becoming infected every timestep (30 seconds). We also investigated the impact of increasing the distance that free virions can travel by allowing the 124 cells (r = 2) nearest a productively infected cell to have a probability of infection at every timestep.

#### Death rate

Since we are interested in the dynamics of CD4+ T cells (uninfected and infected) during chronic HIV infection, we only allow for proliferation and death of CD4+ T cells in the model. The rest of the cell populations remain of constant size during the course of the infection.

For uninfected CD4+ T cells, we set the death rate based on the proliferation rate estimated in [80]. As argued in [95] the death rate measured in [80] is not that of all CD4+ cells but only of labelled cells, which are not a representative sample of the whole. However, the proliferation rate measured is the proliferation rate of the whole CD4+ population. Hence, because at equilibrium the proliferation equals the death rate and since we are mainly interested in the chronic phase of the infection, we set the death rate of uninfected CD4+ T cells to 0.025 d^−1^ which equates to a lifespan of 40 days.

For infected CD4+ T cells, we consider two phases: 1) an eclipse phase (when a cell is infected but not yet producing virions) and 2) a productively infected phase. During the eclipse phase, infected cells can neither be recognised by CD8+ T cells nor infect uninfected CD4+ T cells; they die with the same rate as uninfected cells. The eclipse phase is estimated to last 1 day [96], [97]. In the productively infected phase, cells die at an exponential rate with a mean of 1 d^−1^ (i.e. they have a mean lifespan of 1 day) [98]. This death rate includes death due to all factors (e.g. CD8+ T cell killing, NK cell killing, and viral cytotopathicity).

We set the time to CD8+ T cell appearance to 10 *days*. We set the proportion of productively infected cell death attributable to CD8+ T cells to approximately 30% [28], [48], [49]. The total death rate of productively infected CD4+ T cells is 1 d^−1^, hence, we assume an average death rate of 0.7 d^−1^ before CD8+ T cell activation and an average death rate of 0.95 d^−1^ after activation, allowing a small proportion of the death rate (≈5%) to be attributable to the single epitope-specific CD8+ T cell population that is explicitly included in the model. This implies that the death rate that is not attributed to the total CD8+ T cell population is the same before and after the activation of the CD8+ T cells. To allow for stochastic effects, the lifespan of infected cells, which is the reciprocal of the death rate, is sampled for each infected cell from a normal distribution (mean±sd) of 1.4±0.25 days before introducing CD8+ T cell in the model and 1.05±0.25 days after day 10 (not including the death rate attributable to the explicitly modelled specific-CD8+ T cell population).

### Conjugate formation

We explicitly model the formation of conjugates between the single epitope-specific CD8+ T cell population included in the model and wild-type infected CD4+ T cells. Once a CD8+ T cell encounters a target cell it must make the decision of ‘acting or moving on’; we call the time this takes the *scanning time*. The time that it takes a T cell to scan its target is estimated to be 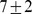 min [41] and this process is explicitly incorporated in the model. In general, the decision to act is related to the characteristics of the TCR-pMHC interaction and the level of pMHC expression on the surface of the target cell [99]. This process is simulated in the model via a probability which defines the decision of the CD8+ T cell upon encounter to which we refer as *probability of recognition* and is discussed below. If the CD8+ T cell recognises the cell as infected then the *interaction time* begins. The interaction time is set to between 10–30 min, in agreement with measurements obtained with intravital multiphoton microscopy [41], [42]. For the lytic model we refer to this interaction time as the “duration of killing”, for the non-lytic models we refer to this interaction time as the “duration of secretion”. The conjugates remain immobile during both scanning and interaction time. This is a good approximation of the underlying biological process; in [41], [56] the conjugates of T cells with B cells and granuloma cells respectively are immobile within minutes of recognition. If the CD8+ T cell fails to recognise that the cell is infected then it will disassociate and move on to another target.

Two additional aspects of the CD8+ T cell and target cell encounter are incorporated in the model: 1) multiple CD8+ T cells can kill the same target [100], [101] and 2) multiple targets can be killed by a single CD8+ T cell as observed *in vitro* [42]. The latter is further supported by the configuration of the contact between TCR and APCs; during recognition of a target cell the microtubule-organising center (MTOC) of the CD8+ T cell polarises towards the immunological synapse. The high motility of the MTOC allows for rapid switch of polarization between targets [102]. However, as found in [101] most of the CD8+ T cells in conjugate formation did not have more than four target cells bound to them, an observation that we implement in the model.

### Target recognition by CD8+ T cells

The effectiveness of CD8+ T cells is dependent on their ability to successfully survey potentially infected cells and recognise them as targets. The recognition is dictated by a series of factors such as: 1) the level of antigenic stimulation [103], [104], 2) the structural rearrangements of TCR-binding [105], 3) the confinement time of the TCR-pMHC interaction [106], 4) the formation of the peptide-MHC complex [107] and others. We simulate the stochasticity of this process by setting a probability of recognition (Pr) of an infected target once the initial scanning time is completed. If the target is successfully recognised then the CD8+ T cell will act (either killing the target if it is operating lytically or secreting soluble factors if it is operating non-lytically). The probability of recognition is one of the parameters which allows us to vary the immune control exerted by the specific CD8+ T cell population included in our simulations.

### Lytic and non-lytic viral suppression

The lytic response can be divided into at least three stages [108]: 1) CD8+ T cells survey potential target cells and recognise a subgroup of them, 2) they then form a conjugate with their target in order to deliver the lethal hit and 3) once the target lyses, they continue hunting for new targets. All these stages are explicitly included in the model. A CD8+ T cell might need to ‘rearm’ before moving on to their next target [109], [110] or pause before identifying a new target [41] therefore increasing the duration of this process (we vary this parameter in order to investigate its effect).

The non-lytic response can be summarised in the following steps: 1) CD8+ T cells survey potential target cells and recognise a subgroup of them, 2) they form a conjugate with their target and 3) they secrete soluble factors which suppress viral production or infection. We model the reduction of infectivity by not allowing the infection of uninfected CD4+ T cells that ‘interact’ with the soluble factors. In a similar way, we model the suppression of viral production by not allowing infected cells to infect other cells once the former ‘interact’ with the secreted factors. The duration of the conjugate is set to 10–30 min as reported in [41]. We simulate the following different secretion patterns: 1) localised effect, where only the wild-type infected cell in conjugate with the CD8+ T cell is affected, 2) a polarised (grid radius, *r* = 1) effect where all the cells one ‘side’ (in grid terms) of the CD8+ T cell are affected and 3) a diffusive pattern where all the cells equidistantly found in the immediate or broader area around the CD8+ T cell are affected by the soluble factor. We considered a number of different radii of diffusion: r = 1 (26 nearest cells affected), r = 2 (124 cells affected), r = 3 (342 cells affected), r = 4 (728 cells affected), r = 5 (1330 cells affected) and r = 6 (2196 cells affected). The duration of the effect depends on the soluble factor under consideration. We focus on RANTES as a case study. The recycling time for CCR5 receptors after interaction with RANTES has a half-life of 6–9 hrs [67] so we set the total duration of the RANTES ‘protective effect’ on uninfected CD4+ T cells to 10 hrs; after that period the uninfected cells can again be infected either with the wild-type or the variant strain. The duration of the protection conferred by the soluble factor is varied to ascertain its impact. In the results presented we consider 100% protection of the uninfected targets unless otherwise stated; reducing the protection to 50% reduced the frequency but not the rate of escape.

For lytic killing, the target cells are lysed at the time that the conjugate is detached. For non-lytic responses, the secretion of soluble factors is stopped after conjugate resolution. This is supported by experimental observations. In [111] it is shown that IFN-γ production from activated CD8+ T cells is terminated immediately after the contact of the T cell and its cognate antigen is disrupted. In the same study [111] , *in vitro* experiments indicate that peptide-pulsed spleen cells cause the production of cytokines (IFN-γ and TNF-α) by LCMV-specific CD8+ T cells within 30 mins. *In vivo*, no cytokine production was observed in the absence of stimulation.

### Quantifying CD8+ T cell killing and viral escape

#### The escape variant

We initiate the simulations with 1–2 wild type-infected cells. Cells infected with the escape variant are introduced at day 50 post infection at a frequency equal to 30% of the wild-type infected cell population unless otherwise stated. Reducing the initial variant frequency to 10% (corresponding to approximately 10 infected cells) reduced the frequency but not the rate of escape. Variant-infected cells cannot be recognised by the single epitope-specific CD8+ T cells but are recognised by all other CD8+ T cells.

#### Estimated viral escape rate

Following [49] the rate of viral escape is estimated by fitting the following equation to the simulated data.
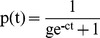
where p(t) is the proportion of variant-infected cells at time t; c is the escape rate and g is the ratio of wild type to variant infected cells when the variant is introduced.

### Simulations

We run the simulation from the time of infection to verify that the model predicts plausible viral expansion. However, we focus on the analysis of the results obtained after the set-point has been attained.

For all the calculations presented in this study, we run a minimum of 10 up to a maximum of 100 simulations for each situation with identical initial conditions and parameter values but different initial seeding of the C++ pseudorandom algorithm.

## Supporting Information

Text S1 Supplementary Text S1 contains the following: Figure S1. Duration of CD8+ T cell killing has little impact on the observed killing rates. Figure S2. The motility of the simulated CD8+ T cells resembles a random walk. Figure S3. The mean CD8+ T cell speed decreases as the probability of recognition of infected targets increases. Figure S4. For CD8+ T cell operating via a lytic mechanism, the proportion of infected cells at set point decreases as the probability of recognition increases. Figure S5. New infections prevented under a non-lytic CD8+ T cell response that blocks infection. Figure S6. Number of infected CD4+ T cells ‘blocked’ from viral production under a non-lytic CD8+ T cell response that blocks production. Figure S7. Set-point of productively infected cells under a non-lytic CD8+ T cell response that blocks production. Figure S8. New infections prevented under a non-lytic CD8+ T cell response that blocks production. Figure S9. Number of uninfected CD4+ T cells ‘protected’ from infection with increasing effector population size. Figure S10. Immune control exerted by a non-lytic response that reduces infectivity. Figure S11. Immune control exerted by a non-lytic response that reduces virion production. Equivalence of non-lytic models in chronic infection. Supplementary methods.
(PDF)
