# Supplementary material for: Can Non-lytic CD8+ T Cells Drive HIV-1 Escape?
Source: PLoS Pathog. 2013 Nov 14;9(11):e1003656. doi: 10.1371/journal.ppat.1003656 (PMC3828169; doi:10.1371/journal.ppat.1003656)
Supplement: Text S1 — Supplementary Text S1 contains the following: Figure S1. Duration of CD8+ T cell killing has little impact on the observed killing rates. Figure S2. The motility of the simulated CD8+ T cells resembles a random walk. Figure S3. The mean CD8+ T cell speed decreases as the probability of recognition of infected targets increases. Figure S4. For CD8+ T cell operating via a lytic mechanism, the proportion of infected cells at set point decreases as the probability of recognition increases. Figure S5. New infections prevented under a non-lytic CD8+ T cell response that blocks infection. Figure S6. Number of infected CD4+ T cells ‘blocked’ from viral production under a non-lytic CD8+ T cell response that blocks production. Figure S7. Set-point of productively infected cells under a non-lytic CD8+ T cell response that blocks production. Figure S8. New infections prevented under a non-lytic CD8+ T cell response that blocks production. Figure S9. Number of uninfected CD4+ T cells ‘protected’ from infection with increasing effector population size. Figure S10. Immune control exerted by a non-lytic response that reduces infectivity. Figure S11. Immune control exerted by a non-lytic response that reduces virion production. Equivalence of non-lytic models in chronic infection. Supplementary methods. (PDF) [file ppat.1003656.s001.pdf]

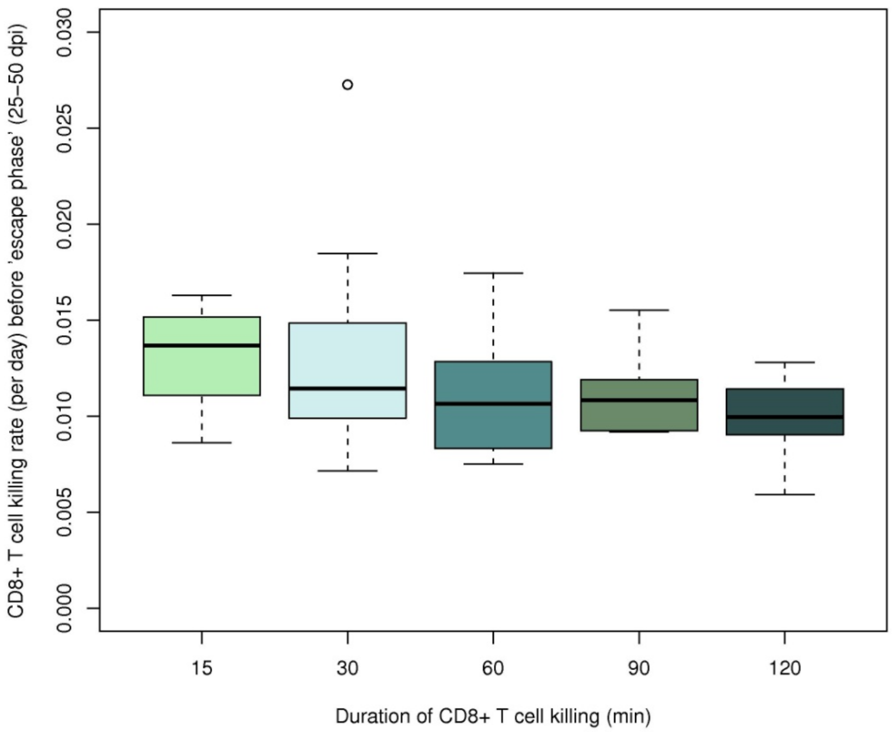

Supplementary Figure S1. Duration of CD8+ T cell killing has little impact on the observed killing rates. The killing rate is calculated for 10-25 simulations in each case and is averaged over 25-50 dpi. The probability of recognition is set to 0.001. We observed a weak trend for the killing rate to decrease as the duration of killing was increased. However, none of the killing rates were statistically significantly different from that observed with a killing duration of 30 mins ($p>0.05$) apart from the case where the killing duration was increased to 120 mins ($p=0.03$); even in this extreme case the mean difference in killing rate was small (0.2%). In a similar model of LCMV infection [1], the authors found that the killing rate depends on the duration of killing however the killing rate in LCMV infection is much faster (as high as 1 min^{-1}) than in HIV/SIV infection (at most 1 day^{-1} [2]). It is therefore not surprising that changing the duration of killing by minutes substantially affects a killing rate of the order of a few minutes but not one of the order of a day; i.e. in HIV-1/SIV the time to successfully find a target rather than the handling time is limiting.

1. Graw F, Regoes RR (2009) Investigating CTL Mediated Killing with a 3D Cellular Automaton. PLoS Comput Biol 5.
2. Markowitz M, Louie M, Hurley A, Sun E, Di Mascio M, et al. (2003) A novel antiviral intervention results in more accurate assessment of human immunodeficiency virus type 1 replication dynamics and T-cell decay in vivo. J Virol 77: 5037-5038.

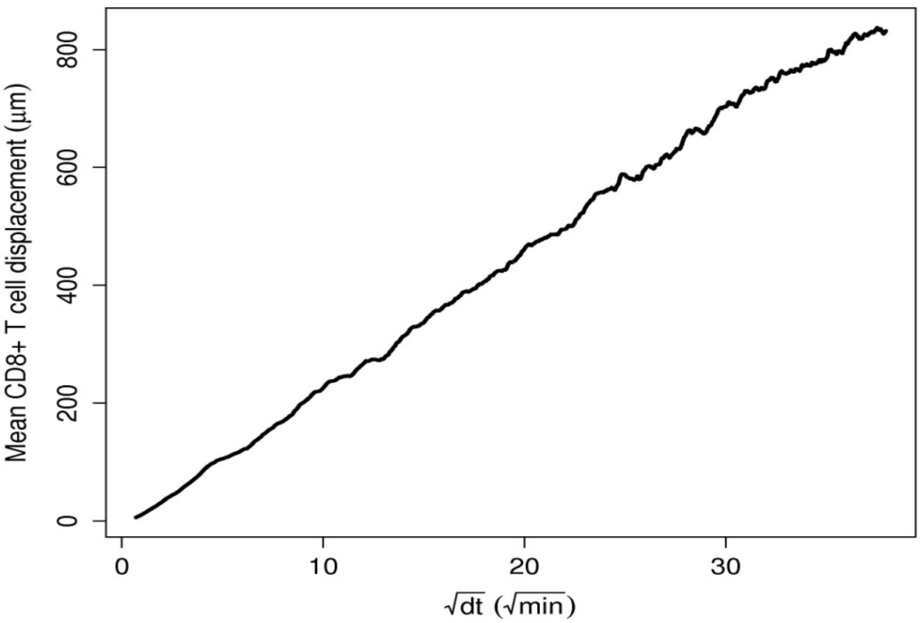

Supplementary Figure S2. The motility of the simulated CD8+ T cells resembles a random walk. The mean displacement of the CD8+ T cells with respect to the square root of time is shown. The straight line suggests that the CD8+ T cell movement resembles a random walk.

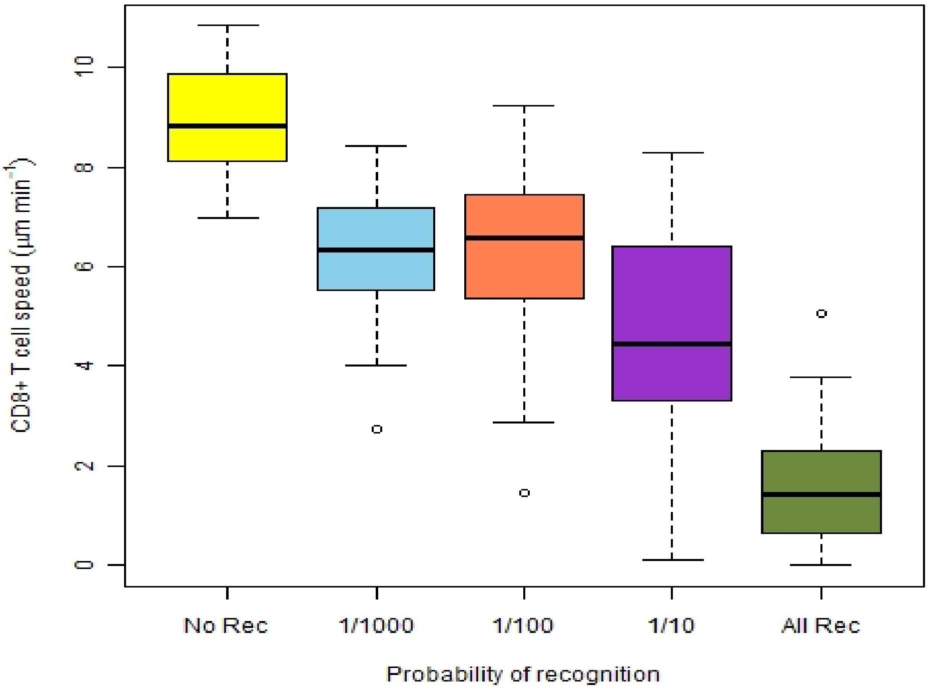

Supplementary Figure S3. The mean CD8+ T cell speed decreases as the probability of recognition of infected targets increases. Each boxplot represents the results obtained by the simulation of 50 epitope-specific CD8+ T cells. Abbreviations: No Rec=CD8+ T cells do not recognise any infected target that they meet, All Rec=CD8+ T cells recognise all infected targets that they meet; the rest represent intermediate cases.

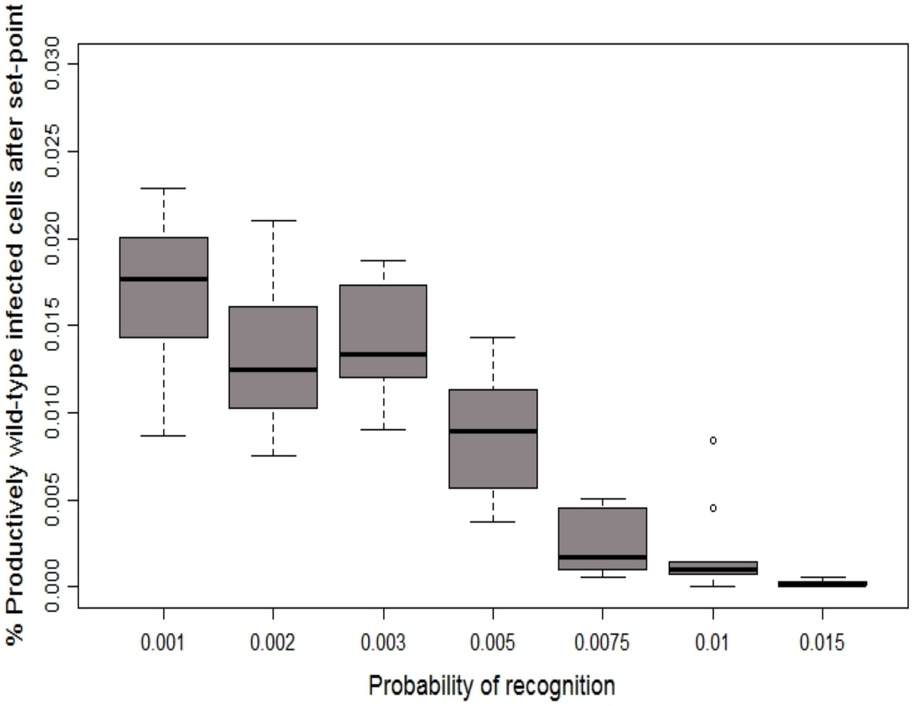

Supplementary Figure S4. For CD8+ T cell operating via a lytic mechanism, the proportion of infected cells at set point decreases as the probability of recognition increases.

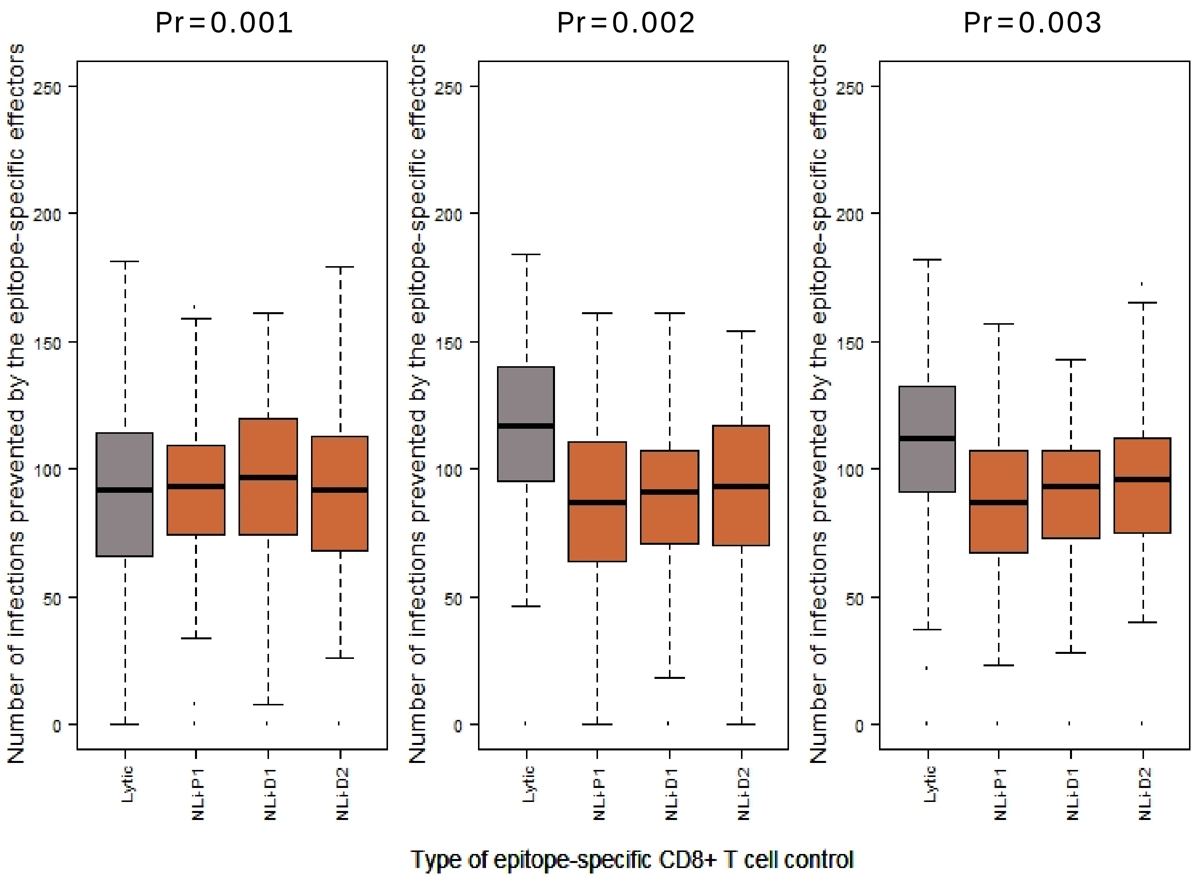

Supplementary Figure S5. New infections prevented under a non-lytic CD8+ T cell response that blocks infection. We show the number of infections prevented by the epitope-specific CD8+ T cell clones for 40-50 dpi, just before the variant infected cell population is introduced in the simulations. Abbreviations: Pr=Probability of recognition, NLi: Non-lytic model - blocking infection of uninfected CD4+ T cells, P1= Polarised secretion (r=1), D1= Diffusive secretion (r=1) and D2= Diffusive secretion (r=2).

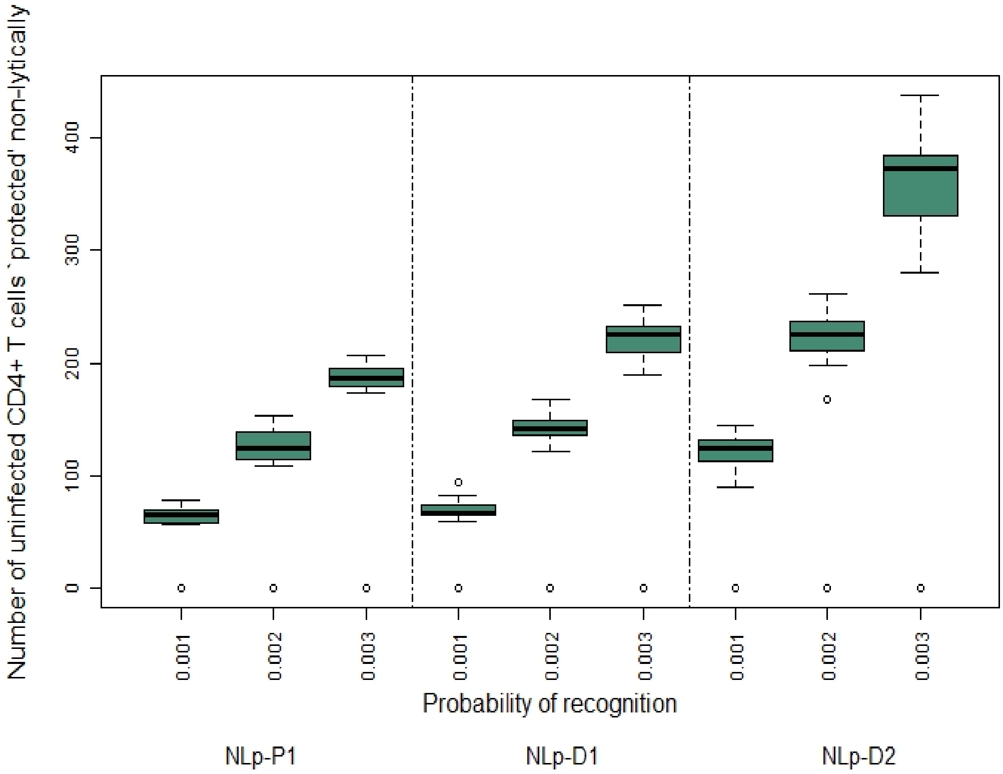

Supplementary Figure S6. Number of infected CD4+ T cells 'blocked' from viral production under a non-lytic CD8+ T cell response that blocks production. The non-lytic CD8+ T cell response is manifested in a polarised or diffusive secretion pattern. Abbreviations: NLp: Non-lytic model - blocking viral production from infected CD4+ T cells, P1=Polarised secretion (r=1), D1=Diffusive secretion (r=1) and D2=Diffusive secretion (r=2).

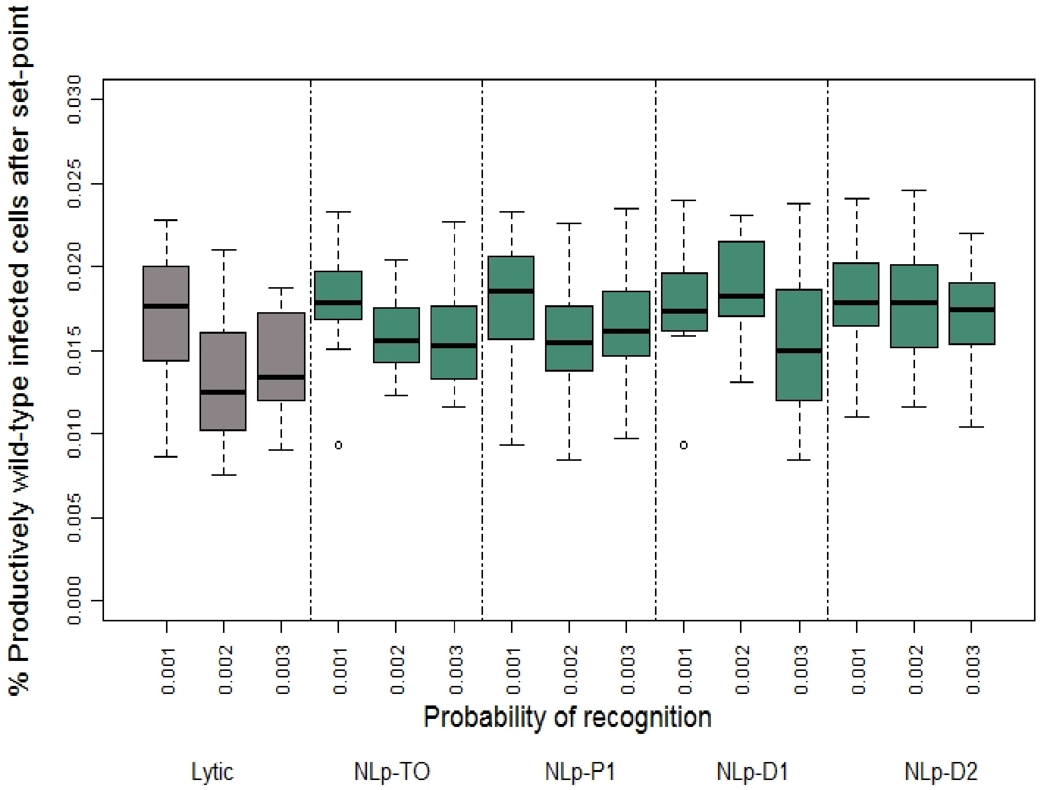

Supplementary Figure S7. Set-point of productively infected cells under a non-lytic CD8+ T cell response that blocks production. We show the percentage of productively infected wild-type cells for 40-50 dpi, just before the variant infected cell population is introduced in the simulations and after the steady-state has been attained. Here, we present the results for the lytic control and the non-lytic control that blocks viral production. Abbreviations: NLp: Non-lytic model - blocking viral production from infected CD4+ T cells, TO= Target Only, P1=Polarised secretion (r=1), D1=Diffusive secretion (r=1) and D2=Diffusive secretion (r=2).

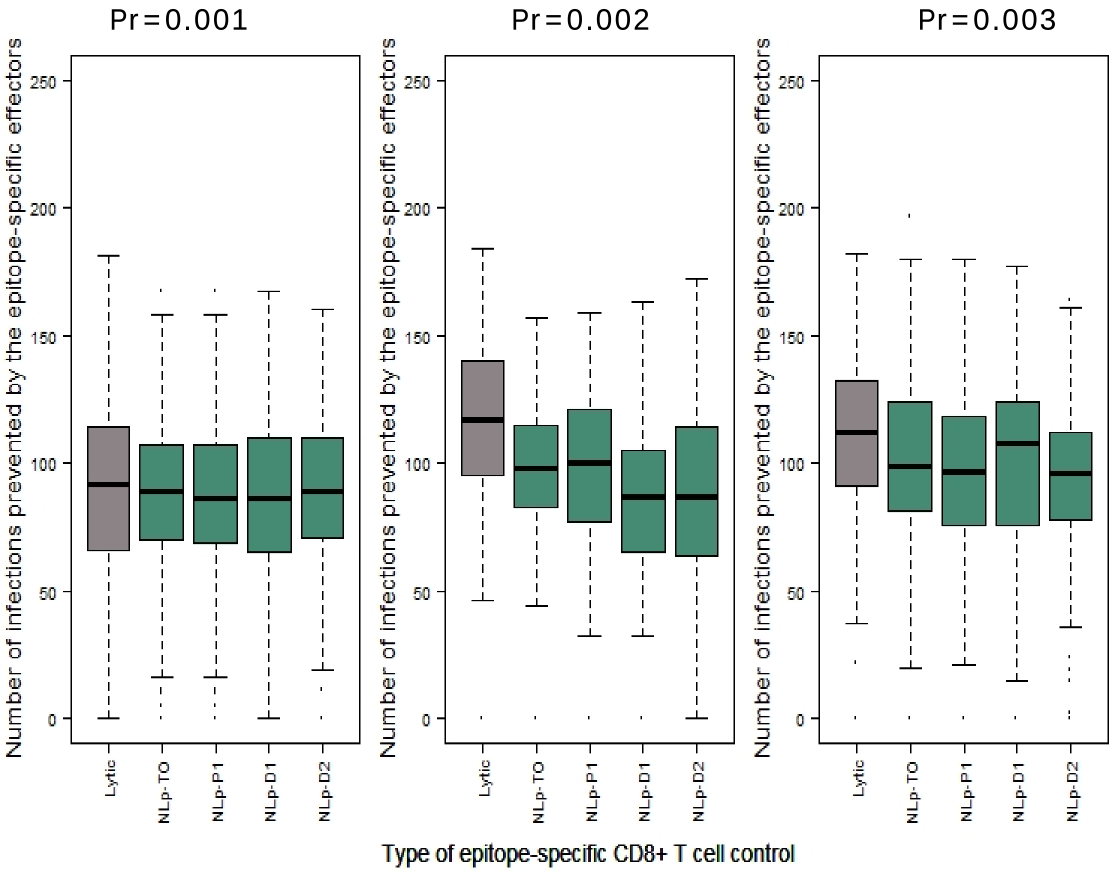

Supplementary Figure S8. New infections prevented under a non-lytic CD8+ T cell response that blocks production. We show the number of infections prevented by the epitope-specific CD8+ T cell clones for 40-50 dpi, just before the variant infected cell population is introduced in the simulations. Abbreviations: NLp: Non-lytic model - blocking viral production from infected CD4+ T cells, TO=Target only, P1=Polarised secretion (r=1), D1=Diffusive secretion (r=1) and D2=Diffusive secretion (r=2).

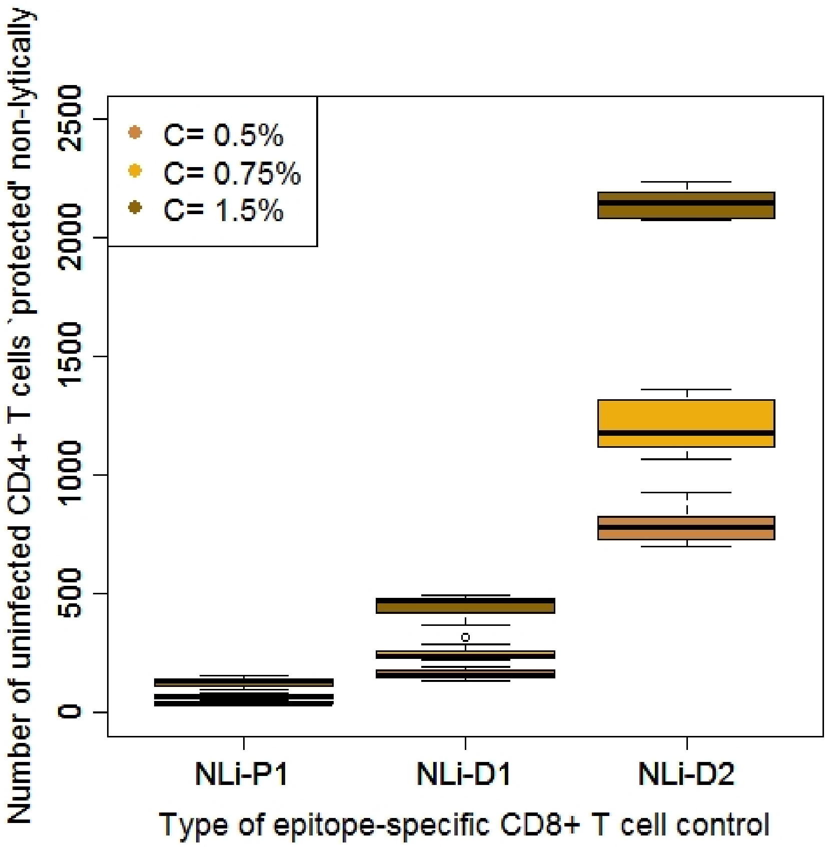

Supplementary Figure S9. Number of uninfected CD4+ T cells 'protected' from infection with increasing effector population size. The CD8+ T cell response is manifested in a polarised or diffusive secretion pattern for different sizes of the epitope specific effector cell population, C, as a percentage of the total splenocyte population. We show the cumulative number at 50 dpi. The probability of recognition is set to Pr=0.002 for all the simulations. Abbreviations: NLi: Non-lytic model - blocking infection of uninfected CD4+ T cells, P1=Polarised secretion (r=1), D1=Diffusive secretion (r=1) and D2=Diffusive secretion (r=2).

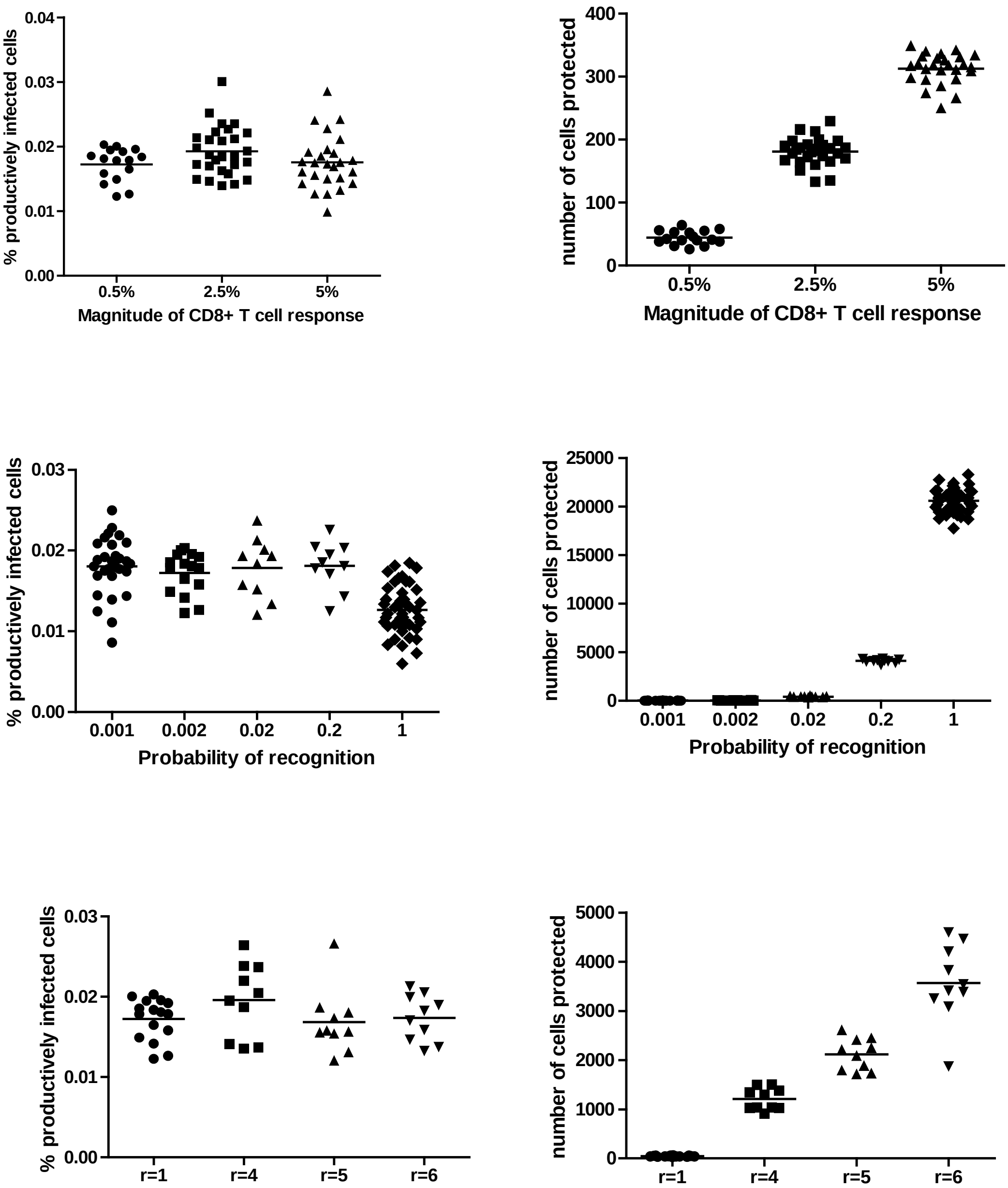

Supplementary Figure S10. Immune control exerted by a non-lytic response that reduces infectivity. Varying the magnitude of the CD8+ T cell response (row 1), the probability of recognition (row 2) and the radius of secretion (row 3) had little impact on the % of infected cells at set point (column 1) despite a large impact on the number of cells protected (column 2). Only in extreme cases e.g. increasing the probability of recognition 1000-fold (row 2) was there a significant impact on % infected cells ($P=3.2\times10^{-9}$, H0: no difference in % infected cells between probability of recognition =0.001 and probability of recognition=1, Wilcoxon Mann Whitney unpaired two-tailed test).

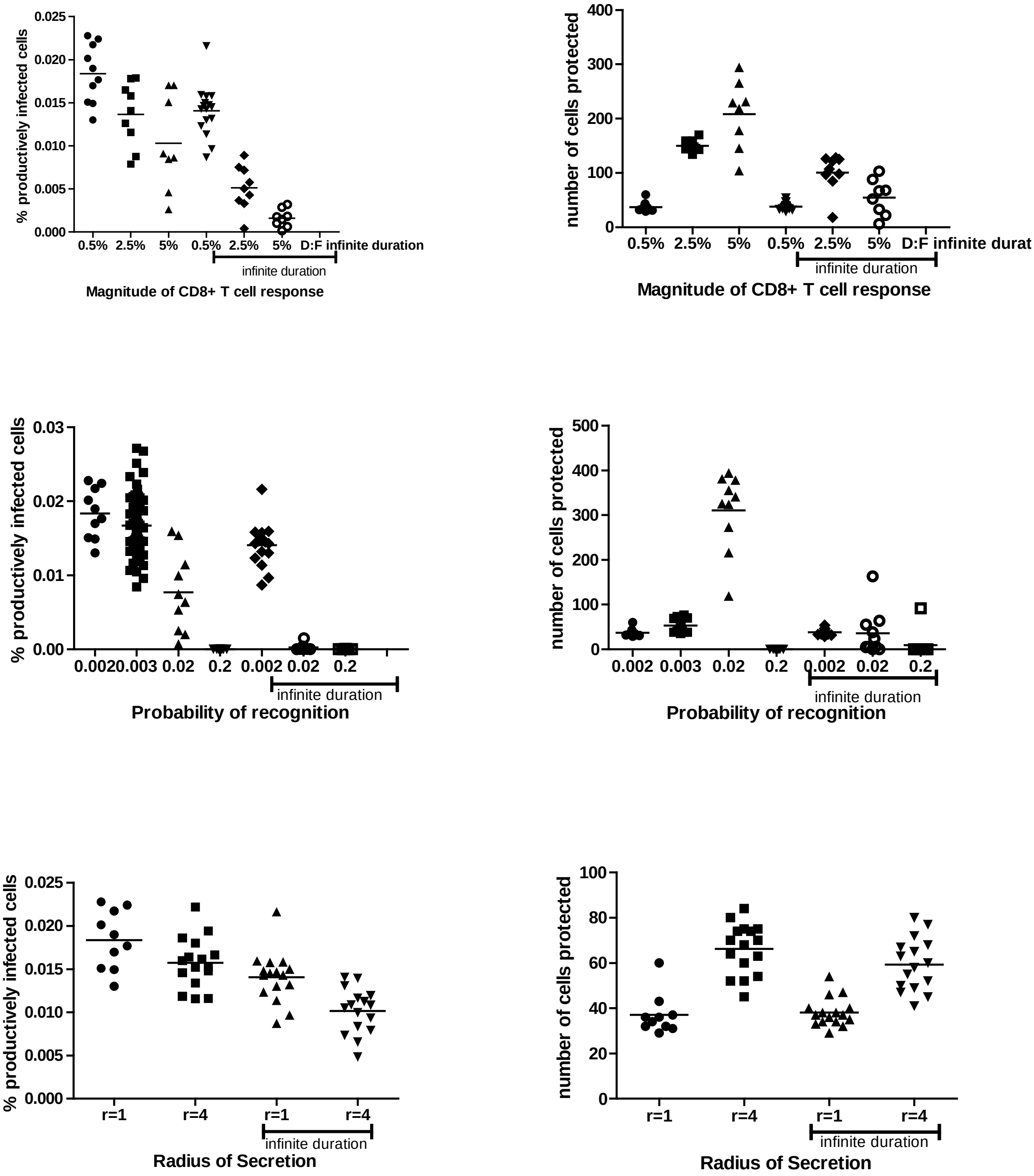

Supplementary Figure S11. Immune control exerted by a non-lytic response that reduces virion production. Parameter changes which readily boost a lytic CTL response (e.g. increasing the probability of recognition from 0.002 to 0.003) had no significant impact on the protection conferred by a non-lytic response that blocks virion production (protection measured as % of infected cells at set point). However, the non-lytic response that blocks virion production (this figure) was considerably easier to boost than the non-lytic response that reduces infectivity (Supplementary Figure S10). In all cases, increasing the duration of the protective effect so that once protected, cells were protected for the duration of the simulation ("infinite duration"), significantly boosted the non-lytic response. P values, H0: there is no difference in the % of productively infected cells; Magnitude 5% v 2.5%: P=0.02. Probability of recognition 0.002 v 0.003: NS. Probability of recognition 0.002 v 0.02: P=0.0003. Radius of secretion r=1 v r=4: NS.

Equivalence of non-lytic models in chronic infection

It can readily be shown that under a quasi-equilibrium between infected cells and free virus which is assumed to hold during the chronic phase of infection, a non-lytic model where the CD8+ T cells reduce viral production and a non-lytic model where CD8+ T cells reduce infection of new targets produce the same dynamics for the population of productively infected cells.

The dynamics of a non-lytic model where viral infection is decreased can be described by

$$\dot{T}^* = \left(\frac{1}{1+\eta E}\right)\beta SV - \delta_I T^* \qquad \text{(1)}$$
$$\dot{V} = pT^* - cV$$

The dynamics of a non-lytic model where viral production is decreased can be described by

$$\dot{T}^* = \beta SV - \delta_I T^* \qquad \text{(2)}$$
$$\dot{V} = \left(\frac{1}{1+\eta E}\right) pT^* - cV$$

where β is the infection rate, *p* is the production rate of free virions, *c* is the clearance rate of free virions, *S* is the number of susceptible target cells, T* is the number of productively infected cells, *V* is the number of free virions, E is the number of virus-specific CD8+T cells, η parameterises the effect of effector CD8+ T cells on the virus and δ_I is the death rate of productively infected cells.

The quasi-steady assumption for the non-lytic model where viral infection is decreased results in:

$$V = \frac{p}{c}T^* \qquad \text{(3)}$$

while the quasi-steady state assumption for the non-lytic model where viral production is decreased results in:

$$V = \left(\frac{1}{1+\eta E}\right)\frac{p}{c}T^* \qquad \text{(4)}$$

Substituting (3) and (4) into the equations governing the behaviour of productively infected cells (T*), (1) and (2) respectively, both equations can be rewritten:

$$\dot{\mathrm{T}}^* = \left[\left(\frac{1}{1+\eta E}\right)\frac{\beta pS}{c} - \delta_I\right]T^* \qquad \text{(5).}$$

Supplementary methods

Initial cell populations			
Quantity	Value	Reference	Notes
Lattice edge	50	n/a	125000 cells, 0.05 – 0.5% of the splenic *white pulp* [1]
Timestep	30 sec	n/a	Integrates micro and macro scale
Reticular network	20%	-	
Free space	1%	-	Spleen is a densely packed organ
Epitope-specific CD8+ T cells	0.5%	[2,3]	Estimated based on ELISPOT assays
CD4+ T cells	15%	-	Mouse spleen
MΦs & DCs	1%	-	

Supplementary Table S1 Initial cell populations. Percentages refer to the number of total grid cells apart from the epitope-specific CD8+ T cell population that is given as a percentage of the splenocytes population.

Influx of infected CD4+ T cells

The CD4+ T cells entering the grid with a probability, p_{influx}, can be uninfected or infected (either with the wild-type or the variant strain). The following equations describe the set of probabilities which define whether the CD4+ T cell that entered the grid will be uninfected, p_u, or infected (with the wild-type, p_w, or the variant, p_v , strain). These probabilities depend on the current population of uninfected, N_u, wild-type infected, N_w and variant infected, N_V, cells present on the grid as well as their respective lifespans, L_u, L_w and L_v. We consider L_w=L_v.

$$p_w = \frac{N_w L_w}{N_u L_u} p_u$$

$$p_v = \frac{N_v L_v}{N_u L_u} p_u$$

$$p_u = \frac{N_u L_u}{N_u L_u + N_w L_w + N_v L_v}$$

This fraction of newly introduced infected cells can represent reservoirs of latently infected CD4 T cells which can be important in maintaining active infection [4,5]. Additionally, it can reflect the small proportion of circulating infected CD4+ T cells that enter the spleen at any given time [6].

1. Barber DL, Wherry EJ, Ahmed R (2003) Cutting Edge: Rapid In Vivo Killing by Memory CD8 T Cells. J Immunol 171: 27-31.
2. Kiepiela P, Ngumbela K, Thobakgale C, Ramduth D, Honeyborne I, et al. (2007) CD8+ T-cell responses to different HIV proteins have discordant associations with viral load. Nat Med 13: 46-53.
3. Frahm N, Yusim K, Suscovich TJ, Adams S, Sidney J, et al. (2007) Extensive HLA class I allele promiscuity among viral CTL epitopes. European Journal of Immunology 37: 2419-2433.
4. Bailey J, Blankson JN, Wind-Rotolo M, Siliciano RF (2004) Mechanisms of HIV-1 escape from immune responses and antiretroviral drugs. Current Opinion in Immunology 16: 470-476.
5. Ramratnam B, Mittler JE, Zhang L, Boden D, Hurley A, et al. (2000) The decay of the latent reservoir of replication-competent HIV-1 is inversely correlated with the extent of residual viral replication during prolonged anti-retroviral therapy. Nature Medicine 6: 82-85.
6. Haase AT (1999) Population biology of HIV-1 infection: viral and CD4+ T cell demographics and dynamics in lymphatic tissues. Annual Review of Immunology 17: 625-656.
